# Changes in the population of patients with dementia referred to Dementia disease centers in Japan as a result of the launch of Lecanemab

**DOI:** 10.1002/alz70858_105340

**Published:** 2025-12-26

**Authors:** Aiko Ishiki, Chika Oyama, Isabelle Miyazawa, Naoki Tomita, Katsutoshi Furukawa

**Affiliations:** ^1^ Tohoku Medical and Pharmaceutical University, Sendai, Miyagi, Japan

## Abstract

**Background:**

In Japan, Lecanemab could be administered to patients with early Alzheimer's disease in September 2023. Since the diagnosis of the disease would need to be made earlier than before, it was expected that patient referrals from primary care physicians to Dementia Disease Center specialists would increase. This study aims to determine if the patient population that visited the Dementia Disease Center changed before and after the launch of Lecanemab in Japanese setting.

**Method:**

Patients visiting our Dementia Disease Center in a city in eastern Japan from January 2022 to August 2024 with a chief complaint of cognitive impairment were included in the study. We analyzed whether there were differences in age, neuropsychological test results, last diagnosis, and severity of cognitive impairment between the periods before and after January 2023, when Lecanemab became available at our hospital.

**Result:**

The total patients examined were 409 (age 79.1±7.4, 62.6% female), 90.3% were diagnosed with dementia and Mild Cognitive Impairment (MCI), 56% were diagnosed with AD and MCI due to AD. There were no significant differences in age ( Before 78.9±7.7 vs. after 79.75±6.5), MMSE (20.8±5.8 vs. 21.0±5.3), or FAST stage (4.1±1.0 vs. 3.9±0.9) between before and after the launch of Lecanemab.

**Conclusion:**

Early diagnosis is important for the administration of anti‐amyloid‐β drugs, although early diagnosis has not increased after the launch of Lecanemab. Facilitated collaboration between primary care physicians and specialists is needed to ensure that the appropriate treatment is given to the appropriate patient.

